# Influence of Nanocomposite PVD Coating on Cutting Tool Wear During Milling of 316L Stainless Steel Under Air Cooling Conditions

**DOI:** 10.3390/ma18091959

**Published:** 2025-04-25

**Authors:** Jarosław Tymczyszyn, Artur Szajna, Grażyna Mrówka-Nowotnik

**Affiliations:** 1 Department of Manufacturing Techniques and Automation, Faculty of Mechanical Engineering and Aeronautics, Rzeszow University of Technology, 12 Al. Powstancow Warszawy Street, 35-959 Rzeszow, Poland; a.szajna@prz.edu.pl; 2 Department of Material Science, Faculty of Mechanical Engineering and Aeronautics, Rzeszow University of Technology, 12 Al. Powstancow Warszawy Street, 35-959 Rzeszow, Poland; mrowka@prz.edu.pl

**Keywords:** 316L stainless steel, wear, cutting forces, surface roughness, nACo3

## Abstract

This study examines the impact of PVD coatings on cutting tool wear during the milling of 316L stainless steel under air cooling conditions. In the experiment, a carbide milling cutter coated with a nanocomposite nACo3 (AlTiSiN) coating was used. The coating was deposited using a next-generation device, the PLATIT π411PLUS, which features one central and three lateral rotating cathodes. The nanocomposite nACo3 coating obtained with this method exhibits exceptionally high structural density and excellent mechanical properties. The new generation of the nACo3 coating demonstrates improved surface properties and a lower friction coefficient compared to previous generations. The findings indicate that PVD nACo3 coatings significantly enhance wear resistance, extending tool life while maintaining acceptable surface quality. The optimal cutting time was determined to be approximately 90 min, after which a sharp increase in surface roughness and tool wear was observed. After 120 min of machining, substantial deterioration of surface quality parameters was recorded, suggesting increasing cutting forces and cutting edge degradation. SEM and EDS analyses revealed the presence of adhered material on the tool and sulfide inclusions in the microstructure of 316L stainless steel, which influenced the machining process. The nACo3 coating demonstrated high thermal and wear resistance, making it an effective solution for machining difficult-to-cut materials. This study suggests that selecting appropriate cutting parameters, tool geometry, protective coatings, and cooling strategies can significantly affect tool longevity and machining quality. The novelty of this research lies in the application of innovative nanocomposite PVD coatings during the milling of 316L stainless steel under air cooling conditions. These studies indicate potential future research directions, such as the use of minimum quantity lubrication (MQL) or cryogenic cooling as methods to reduce tool wear and improve post-machining surface quality.

## 1. Introduction

Due to the continuous improvement of semifinished product manufacturing technologies, the development of cutting tool materials, and advancements in modern manufacturing machinery, dry machining is becoming an increasingly desirable production method from both ecological and economic perspectives. The elimination of cutting fluids reduces production costs and has a significant impact on environmental protection [1]. Dry machining offers numerous advantages but also presents certain challenges. The primary benefits include improved cleanliness of machined surfaces, reduced costs associated with chip recycling (due to the absence of oil contamination), and lower expenses related to cutting fluids [2]. To fully exploit the potential of dry machining, extensive research and development efforts are required to minimize excessive tool wear [3,4,5,6,7,8].

As early as 2001, within the framework of the National Nanotechnology Initiative in the United States, efforts were initiated to develop coatings and nanostructured materials specifically designed for cutting tools used in dry machining [9].

Tungsten carbide, widely utilized in the production of cutting tools, is the most commonly employed material for machining operations. During machining, cutting tools are exposed to various wear mechanisms. For instance, in dry machining of commonly used 1.0503 steel, the cutting zone temperature can reach between 600 and 950 °C, depending on the cutting speed, while even higher temperatures are observed when machining stainless steels. Under these conditions, cutting tools are particularly susceptible to wear caused by adhesion and diffusion processes, especially when temperatures approach 900 °C (for tungsten carbides) or 1000 °C (for tungsten carbides with titanium) [10].

The application of PVD coatings significantly enhances the performance of cutting tools, particularly in the machining of stainless steel. Carbide tools coated with AlCrN exhibit nearly twice the tool life and improved surface finish compared to uncoated tools [11].

TiAlN coatings, especially when applied in multiple layers, demonstrate the lowest wear rates [12].

The integration of coating design and substrate material is crucial for optimizing tool performance. Both CVD (TiCN + Al_2_O_3_) and PVD (AlTiN) coatings on cemented carbide substrates are effective in machining austenitic stainless steel, with coating thickness and substrate material playing a significant role in tool longevity and wear resistance [13].

Post-processing of PVD-coated surfaces, such as drag finishing and abrasive jet machining, can improve coating surface quality by reducing droplets formed during the PVD deposition process (which involves evaporating solid material in a vacuum and depositing it onto the component surface) and enhancing wear resistance [14].

Overall, PVD coatings substantially improve cutting tool durability and the machining quality of 316L stainless steel under various material removal conditions.

The exceptional properties of 316L stainless steel, including its biocompatibility and distinctive physical, mechanical, and biological characteristics, have led to its increased application across various industries, particularly in the biomedical sector over the past 50 years. 316L stainless steel is widely used in welding, where it provides optimal corrosion resistance. However, machining stainless steel using conventional methods presents certain challenges. The machining of 316L stainless steel has been investigated from various perspectives to enhance efficiency and minimize tool wear. Wire electrical discharge machining (WEDM) has proven effective for complex geometries [15]. The application of minimum quantity lubrication (MQL) has resulted in reduced tool wear compared to dry machining [16]. The study presented in [17] examined the influence of tungsten carbide (WC) on wear resistance, cutting temperature, and surface quality during the milling of 316L stainless steel.

The literature contains numerous studies on cutting forces and temperatures, primarily in the context of turning [18,19]. Umbrello et al. [20] present a study on the influence of different sets of material constants in the Johnson–Cook (J–C) constitutive equation on the finite element modeling of orthogonal turning of 316L stainless steel, examining their impact on experimental and predicted cutting forces, chip morphology, temperature distributions, and residual stresses. The authors observed that all considered process outputs, particularly residual stresses, are highly sensitive to the J–C material constants. Arrazola et al. [21] proposed a novel approach to friction identification in machining through finite element modeling. This approach involves applying a variable friction coefficient at the tool–chip interface, which enhances the agreement between numerical results and feed force measurements.

Uysal et al. [22] investigated surface roughness and chip formation during milling of stainless steel using the minimum quantity lubrication (MQL) method with the commercial vegetable oil Eraoil KT/2000 as a cooling–lubricating fluid. The highest surface roughness was observed during dry cutting. Surface roughness could be reduced by applying a TiN coating; however, the lowest surface roughness was measured during milling with MQL cooling. When analyzing chip shapes, more deformed and irregular chip forms were observed during dry milling. The TiN coating and MQL method resulted in more regular and acceptable chip shapes. Surface roughness was reduced due to effective lubrication and cooling with the MQL method, as reported in the literature [23,24].

Lv et al. [25] evaluated the effect of different cutting edge geometries on the milling of 316L stainless steel using the finite element method. They analyzed a symmetric cutting edge geometry (K = 1, rounded edge) and asymmetric geometries (K = 0.5, waterfall edge; K = 2, trumpet edge) in solid carbide milling cutters. Their study examined plastic deformation, residual stresses, and temperature distribution. The tool–chip contact length and the effective rake angle (γ) were also assessed.

Grabowski et al. [26] demonstrated that small variations in the cutting edge radius significantly affect cutting forces and surface roughness, with this effect varying depending on the helix angle of the tool’s cutting edge. Edge preparation techniques can increase the cutting edge radius and smooth the cutting edge, leading to reduced cutting forces and improved surface finish, particularly when combined with minimum quantity lubrication [27].

Ozcelik et al. [28] investigated the cutting performance in dry and wet conditions during face milling of 316L stainless steel. The experiment aimed to compare tool wear, milling force components, and surface roughness under different operating conditions. The results of the experimental study confirmed that the presence of a semi-synthetic cutting fluid negatively affects the milling of 316L stainless steel. Catastrophic tool failure during wet milling may result from intense thermal stress induced by the application of the semi-synthetic cutting fluid.

Ciftci [29] examined cemented carbide tools with multilayer coatings (TiC/TiCN/TiN and TiCN/TiC/Al_2_O_3_) applied via chemical vapor deposition (CVD) during dry turning of 304 and 316L stainless steel, focusing on surface roughness and cutting forces. Based on the results, he concluded that TiC/TiCN/TiN-coated cutting tools generate lower cutting forces than TiCN/TiC/Al_2_O_3_-coated tools. He also found that 316L stainless steel exhibits higher cutting forces than 304 stainless steel, which was attributed to the presence of 2% molybdenum.

Table 1 presents a comparison of coatings used on cutting tools intended for machining stainless steels.

Surface quality after machining is a key parameter in mechanical engineering and materials science. Surface roughness affects the durability and operational performance of components. Factors such as cutting time, tool wear, and machining temperature significantly influence the achieved surface quality.

This study investigated the influence of a PVD coating on the wear of a cutting tool during the milling of 316L stainless steel under air-cooling conditions. The experiment was conducted using a carbide end mill coated with a nanocomposite nACo3 (AlTiSiN) coating. The results indicate that this coating significantly enhances wear resistance, extending tool life while maintaining an acceptable surface quality. The nACo3 coating exhibited high thermal and wear resistance, making it effective for machining hard-to-cut materials.

Despite extensive research on the use of PVD coatings in machining, most previous studies have been limited to conventional coatings (e.g., TiN, TiAlN) or focused on general tool life behavior without simultaneously addressing surface quality and wear mechanisms. Furthermore, there is a noticeable gap in studies evaluating the performance of modern nanocomposite coatings like nACo3 (AlTiSiN) in the machining of austenitic stainless steels, especially 316L. This paper aims to fill this gap by providing a comprehensive analysis of tool wear and surface roughness as a function of cutting parameters, offering new insights for industrial applications.

The novelty of this research lies in the evaluation of a less commonly studied nanocomposite PVD coating nACo3—(AlTiSiN) during the dry machining of 316L stainless steel, a material known for its difficult-to-machine properties.

Unlike most prior studies, which focus on more conventional coatings such as TiN, TiAlN, or multilayer systems, this work demonstrates the effectiveness of the nACo (AlTiSiN) nanocomposite in reducing tool wear and improving surface finish.

Furthermore, the integration of surface roughness analysis, flank wear monitoring, and SEM/EDS characterization offers a holistic understanding of the coating performance under various machining conditions.

The outcomes of this research provide actionable insights for industrial applications aiming to enhance machining efficiency and tool durability.

Future studies may explore the use of minimum quantity lubrication (MQL) or cryogenic cooling to further reduce tool wear and improve surface quality.

## 2. Materials and Methods

This section describes the configuration of the experimental setup, the machining process used, as well as the equipment and measurement methods applied in this study.

### 2.1. Work Material

In this study, austenitic stainless steel AISI 316L was selected as the workpiece material due to its widespread application across various industries and its poor machinability. The test samples were blocks with dimensions of D300×100 mm. The fundamental mechanical properties of the steel and its chemical composition are presented in Table 2 and Table 3, respectively [31].

### 2.2. Milling Tools

The tool used in the tests is a carbide end mill with a diameter of 12 mm, a cutting edge length of 26 mm, and an overall length of 82 mm. The geometric details are presented in Table 4. The end mill was manufactured using a 5-axis grinding method from a solid WC–Co sintered carbide rod, grade MK12 (Table 5). Prior to the application of protective coatings in PVD processes, the tool underwent an additional technological procedure involving edge rounding to enhance tool durability. A drag finishing process was performed for the tested tool. The drag finishing process was carried out on the DF-5 machine from OTEC. The applied media with loose abrasive consisted of walnut shell granules impregnated with polishing paste. During this process, the tools rotate around their own axis, for example, for 5 min in the clockwise direction and 5 min in the counterclockwise direction. This ensures a fully repeatable and uniform cutting edge with a rounding accuracy of ±1 µm. To verify this value after the production process, the cutting edge radii were measured using an Alicona Infinite Focus microscope.

The tool used in the tests was coated with an nACo3 coating produced in a PLATIT π411PLUS device using cathodic arc deposition (ARC-PVD, where PVD stands for Physical Vapor Deposition). The nACo3 coating was applied according to the technology developed by PLATIT. This type of coating is recommended for, among other things, machining stainless steels (Table 6).

### 2.3. nACo3 Coating Characterization

The nACo3 coating, with a nanocomposite structure based on AlTiSi, was designed for cutting applications such as milling and drilling of hard materials, including hardened tool steels, stainless steels, and heat-resistant alloys. Due to its advanced multilayered structure, the nACo3 coating ensures excellent adhesion to tools and high performance, even in more demanding processes such as milling with ceramic-coated tools and tools made from cubic boron nitride (CBN).

The nACo3 coating consists of a multilayer structure of TiN–AlTiN–AlTiN/Si_3_N_4_, where:

TiN provides high hardness and wear resistance.
AlTiN increases thermal stability and oxidation resistance.
Si_3_N_4_ minimizes friction and enhances wear resistance under high-temperature conditions.

The nACo3 coating is particularly effective in machining materials that require precise control of heat and friction, making it an ideal solution for advanced cutting processes where both tool durability and high-quality machining are required.

The silicon (Si) content in the coating depends on the deposition rate, which can be precisely controlled during the PVD (Physical Vapor Deposition) process or similar techniques. For the Ti–Si–Al–N coating, Si and Al ions replace Ti in the crystal lattice of c-TiN (cubic TiN), leading to a reduction in the crystal lattice parameters of the coating. This change impacts the mechanical and thermal properties of the coating, such as hardness, wear resistance, and stability at high temperatures [32].

The nucleation process and segregation of the amorphous Si_3_N_4_ phase in the coating, with an appropriate silicon content, inhibit the grain growth of nanocrystalline nc-(Ti, Al)N. This results in the formation of ultra-small nanocrystalline AlTiN grains, which are deposited in an amorphous Si_3_N_4_ matrix [30,33].

The grain growth inhibition effect increases the hardness and stability of the coating. Furthermore, the presence of the Si_3_N_4_ phase, which is chemically stable and resistant to high temperatures, contributes to improved oxidation and wear resistance, even under extreme conditions. Due to this advanced structure, Ti–Si–Al–N coatings are used in cutting tools such as end mills, drills, and turning inserts, particularly in the machining of hard materials like stainless steel, heat-resistant alloys, and hardened tool steels [34,35].

### 2.4. Milling Process

The machining process involved end milling of a block of 316L stainless steel. Subsequent experimental steps (n) included the removal of a material layer with a width of ae = 1 mm and depth of ap = 18 mm at a constant cutting speed vc = 120 m/min and feed per tooth fz = 0.06 mm/tooth, where z is the number of teeth in the tool. Compressed air at a pressure of 6 bar was used as the cooling medium, supplied through nozzles located on the spindle housing of the milling machine.

### 2.5. Test Stand, Measurement Methods

The basic test stand was built based on a multi-axis DMU 100 monoBLOCK milling center.

**Figure 1:**
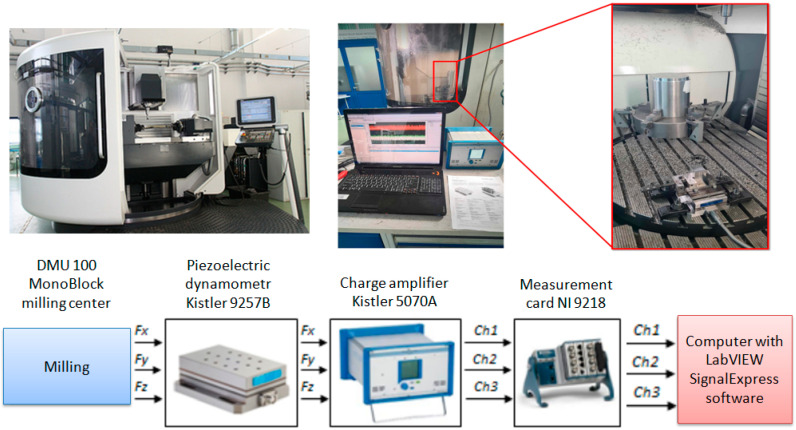
Experimental setup for cutting force measurement using the Kistler 9257B sensor integrated with the data acquisition system.

The cutting forces were measured using a piezoelectric force sensor, Kistler 9257B (Winterthur, Switzerland), with a measurement range of ±5 kN, mounted on the machine table (Figure 1). The signal from the sensor is transmitted to a charge amplifier and sent to a computer via a USB connection using a 16-bit analog-to-digital converter with a measurement range of ±10 V. Signal visualization, processing, and recording were performed using a program developed in the LabVIEW environment. The sampling frequency was set to 20 kHz.

**Figure 2:**
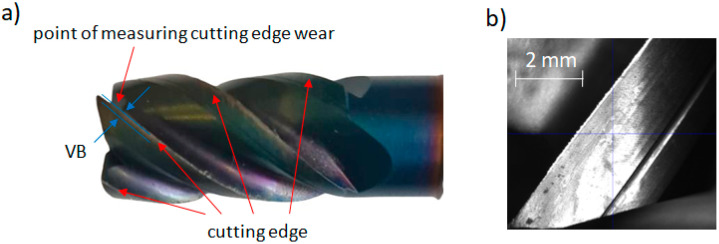
(**a**) Method for determining flank wear width VB on the cutting tool; (**b**) Cutting edge condition at 100× magnification after initial machining.

Tool wear was measured using a DinoLight AM7915MZT microscope (Torrance, CA, USA) combined with a Zoller Genius measuring device (Ann Arbor, MI, USA). The parameter used to evaluate tool wear was the flank wear width VB (mm), which is commonly used to estimate the cutting capability of the tool [36,37]. The method for determining VB and a sample microscopic image are shown in Figure 2.

**Figure 3:**
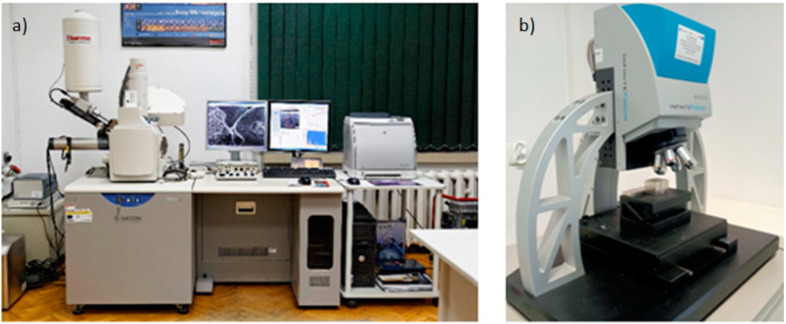
(**a**) Scanning Electron Microscope S-3400N HITACHI (SEM) used for microstructure analysis and (**b**) Alicona Infinite Focus G4 microscope for surface topography and roughness evaluation.

Surface observations of 316L stainless steel after cutting, with different tool wear times (0, 30, 60, 90, and 120 min), were conducted using a scanning electron microscope (SEM) S-3400N HITACHI (Tokyo, Japan) with an Energy-Dispersive X-ray Spectroscopy (EDS) system for microanalysis (Figure 3a). Surface morphology analysis of the stainless steel after machining was carried out in secondary electron (SE) mode. For the observation of worn tools, backscattered electron (BSE) mode was used, allowing phase and material contrast analysis. The EDS system enabled qualitative elemental analysis at selected points and areas on both the machined steel surface and the worn cutting tool. The combination of SEM observations with EDS microanalysis allowed for the detection of any inclusions, contaminations, or other defects.

Surface roughness measurements after machining included parameters Sa, Ra, and Rz. Additionally, surface maps were made to analyze structural changes. The measurements were performed using an Alicona Infinite Focus G4 microscope (Graz, Austria) (Figure 3b).

## 3. Results and Discussion

**Figure 4:**
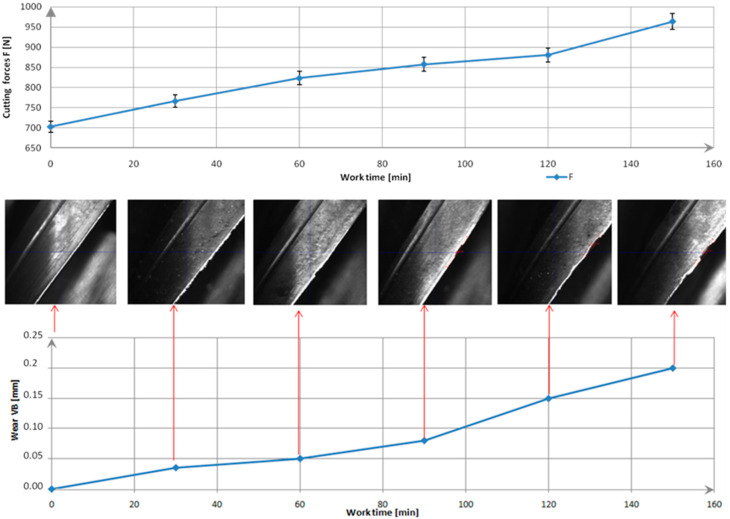
Shows Cutting edge wear development (VB) at 100× magnification and progression of total cutting force (F) over machining time.

In the first phase (0 min) of this study, the tool was new. The total cutting force F was about 700 N. After 30 min of operation, the first signs of microscopic wear (abrasive wear) appeared. Minor damage and material adhesion were visible on the coating. Wear began, but its impact on the cutting force was still negligible. Clear signs of wear on the edge were observed after 90 min. The total cutting force increased by about 22% from the start of the tests. Advanced wear of the cutting edge was evident after 120 min. Numerous chippings and traces of adhered material (adhesive wear) were visible. The flank wear VB reached approximately 0.15 mm, and the total cutting force increased to around 885 N. A VB of 0.2 mm was achieved after 150 min, with the total cutting force reaching 972 N (Figure 4). A typical progression of tool wear was observed throughout the tool’s life cycle: initially small changes, followed by accelerated wear and eventually critical damage. These phenomena were also observed and described in the literature, where similar tool wear patterns were reported [37,38]. The study was repeated for five tools manufactured within the same production batch. No significant differences were observed. The values of the cutting force components and the condition of the cutting edges were comparable, falling within a 5% measurement uncertainty.

**Figure 5:**
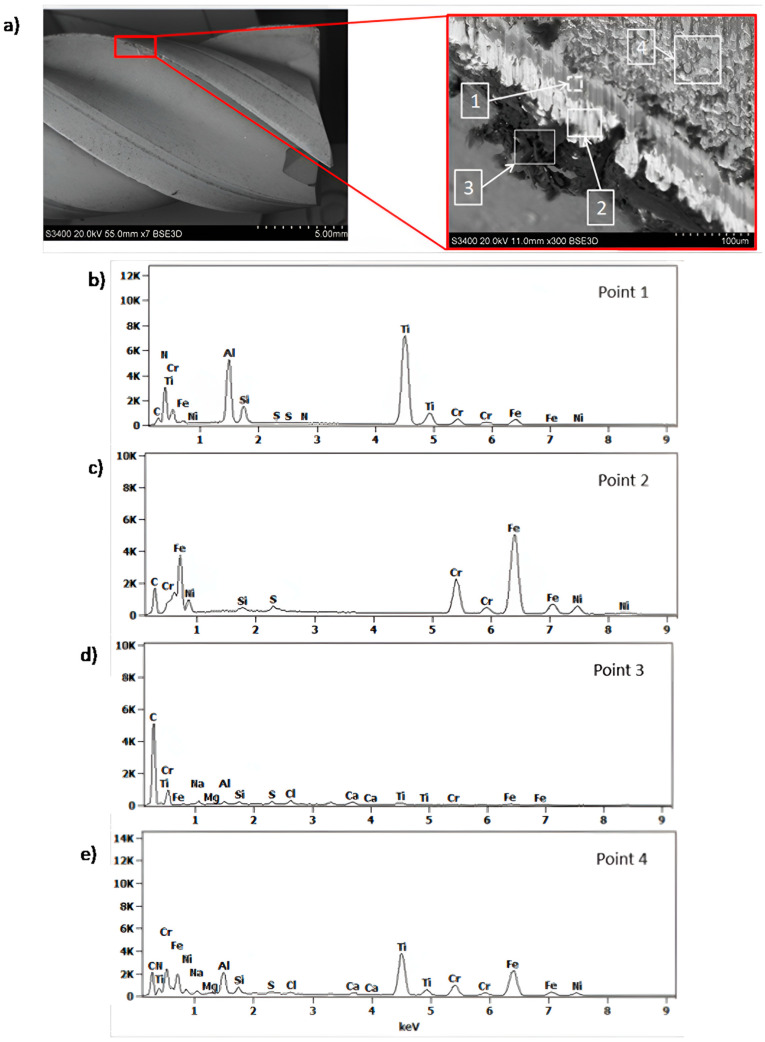
Results of surface and chemical composition analysis of the multilayer nACo3 coating after 120 min of cutting: (**a**) view of the studied surface, (**b**–**e**) EDS spectra at selected characteristic points showing presence of coating components and adhered material from the workpiece.

The chemical composition analysis of the coating and the generated build-up after machining 316L stainless steel is shown in Figure 5. At point 1, the presence of nitrogen (N), silicon (Si), and metals such as chromium (Cr), aluminum (Al), and titanium (Ti) was confirmed, which are components of the multilayer coating nACo3 (Figure 5b). In the analysis of the generated build-up (point 2), the presence of elements like carbon (C), silicon (Si), sulfur (S), chromium (Cr), nickel (Ni), and iron (Fe) was confirmed, indicating the presence of the workpiece material 316L stainless steel (Figure 5c). Locally, a dark build-up (point 3) was observed, which may indicate overheating due to high cutting temperatures (Figure 5d). At point 4, traces of adhered material were observed on the coating surface (Figure 5e).

The surface of 316L stainless steel after machining exhibits parallel tool marks—grooves and waves—resulting from the motion of the cutting tool. Surface microstructure analysis was carried out using a scanning electron microscope (SEM) at two different magnifications: 100× and 600×. The lower magnification allowed for observation and evaluation of the general condition of the machined surface over a larger area, while the higher magnification enabled detailed analysis of local damage. SEM images taken at both magnifications for the surface after machining with a new tool (tool life “0”) as well as for samples machined for 30, 60, 90, and 120 min are presented in Table 7, illustrating both the overall surface characteristics and the details of damage formed on the machined 316L stainless steel surface. Observations made with a scanning electron microscope (SEM) revealed that the surface quality of the material after machining with a new tool—tool life “0”—is worse compared to the surfaces of samples after 30 and 60 min of tool use. This could be influenced by several factors. In the initial stage of tool operation, this is natural and results from the initial mating of the contact surfaces. The phenomenon occurring here is described in publication [39], where the authors explain various mechanisms of tool run-in. The next period of operation corresponds to low wear intensity, characteristic of the normal tool life stage. The best surface quality was achieved for tool usage times of 30 and 60 min. Extending the machining time to 90 min caused an increase in roughness, deformations, grooves or scratches, and localized discoloration of the material due to excessive friction and high temperature in the cutting zone—burning or overheating. The worst surface quality was observed after the longest cutting time, i.e., 120 min, which may result from wear of the cutting edge (Table 7). In the last period, the intensity of cutting edge wear increases rapidly. Working in this time period (90–120 min) becomes essentially unprofitable, as little is gained from the tool life extension, but a lot is lost due to significant wear and surface quality deterioration after machining. These mechanisms were also described in a similar manner in publication [40], and the authors drew similar conclusions regarding tool wear and its impact on the quality of the machined surface.

A comprehensive set of surface topography measurements was conducted, with each sample being measured five times under consistent measurement conditions. A statistical analysis was performed for each surface roughness parameter to determine the mean value, standard deviation, and expanded measurement uncertainty. These calculations were conducted assuming a 95% confidence level, in accordance with Student’s *t*-test.

Surface images presented in Table 8 show differences in topographical structure at various cutting times. As the cutting time increases, changes on the surface due to tool wear become apparent. At the beginning of machining, the tool is sharp and ensures efficient material removal. The best surface quality was achieved after 60 min of cutting, where Sa reached its lowest value of 0.588 µm, and other parameters also indicated acceptable surface quality. Further cutting resulted in an increase in roughness parameters. The increase in temperature after 90 min can cause changes in microstructure and increased cutting forces, leading to process instability. The cutting edge of the tool starts to round off and wear out. The cutting process begins to generate more heat, causing local deformations and increased cutting forces. There may be the onset of a “pulling” effect, rather than precise cutting, which increases roughness. After 120 min, roughness parameters increased by more than 50%, indicating tool wear and deterioration in machining quality. Severe tool wear can cause material adhesion to the tool (Build-Up Edge, BUE), further worsening surface quality. Authors in the publication [41] presented the process of tool wear during milling of stainless steel. It was shown that the average surface roughness increases with the degree of tool wear, which aligns with the results obtained and described above.

**Figure 6:**
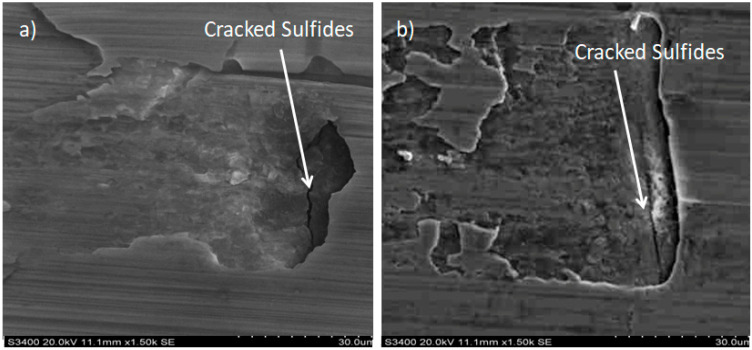
SEM images showing cracked sulfides on the machined 316L stainless steel surface: (**a**) after 30 min and (**b**) after 60 min of machining.

More detailed observations showed that regardless of tool usage time, various defects appeared on the 316L stainless steel surface after machining, with the highest number—as previously mentioned—occurring after the longest tool usage time, i.e., 120 min. In addition to plastic surface deformations, scratches, and nicks, another commonly observed defect was the presence of very hard sulfide inclusions, which cracked under cutting force during contact with the tool, leaving defects on the machined surface (Figure 6).

**Figure 7:**
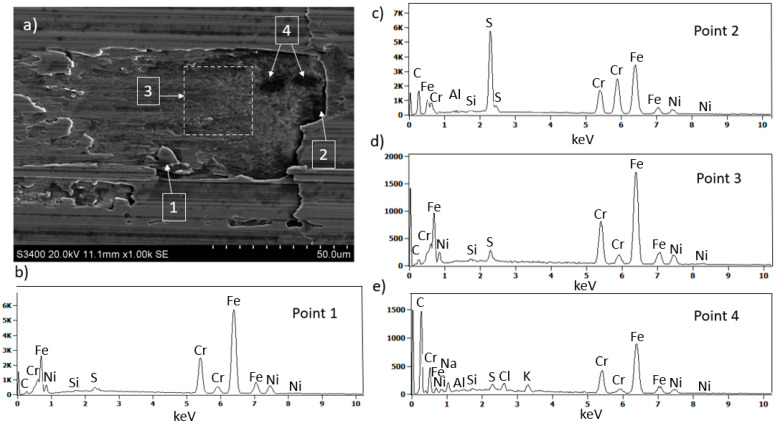
SEM image of the 316L stainless steel surface after 120 min of machining (**a**) and corresponding EDS spectra in marked regions showing chip adhesion (**b**), sulfide damage (**c**), sulfur distribution (**d**), and localized burn mark (**e**).

SEM surface observations of 316L stainless steel after machining (Figure 7a), combined with EDS microanalysis, showed that as a result of tool contact with the machined surface, chip adhesion occurs (point 1 Figure 7a,b). Moreover, due to tool contact with hard sulfides (point 2 Figure 7a,c), surface damage occurs, and sulfur is drawn onto the damaged steel surface (area 3 Figure 7a,d). On the damaged surface of 316L stainless steel near the sulfides, black “spots” were also observed (point 4 Figure 7a,e). Black areas on the machined material surface may indicate local burn marks.

**Figure 8:**
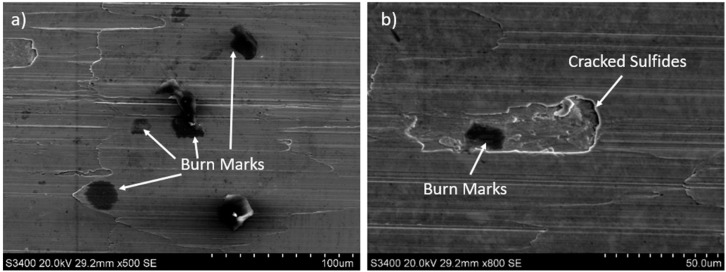
Burn marks on 316L stainless steel surface: (**a**) at the initial stage of machining (tool life 0 min), (**b**) after 30 min of machining.

Observations of 316L stainless steel at the initial stage (Figure 8a) and after 30 min of machining revealed that the local accumulation of sulfides may contribute to burn marks (Figure 8a,b). Similar findings were observed on other surfaces of 316L stainless steel after longer tool operation times. The causes of burn marks on the machined surface of stainless steel could be

- Excessive heating of the steel surface due to inadequate cooling;
- Excessively high cutting speed or large tool force applied to the material surface;
- A dull tool or one with improper geometry, which can increase friction and consequently raise the temperature;
- A high sulfur content or other additives.

**Figure 9:**
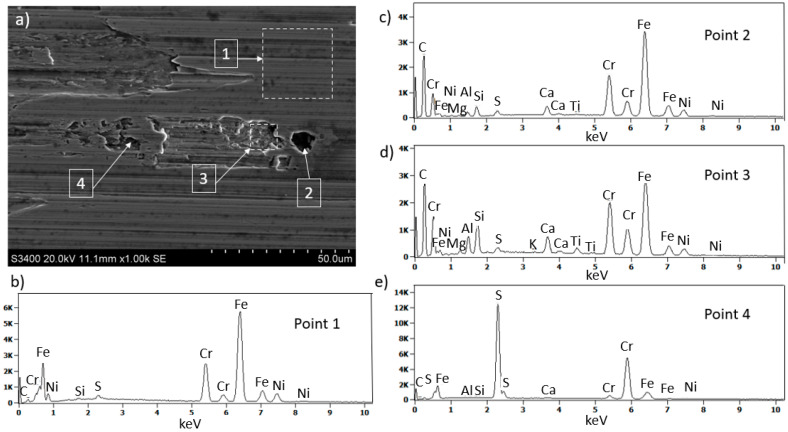
SEM image and EDS analysis of the machined surface after 120 min: (**a**) general view, (**b**–**e**) EDS spectra showing carbide precipitations, coating elements, and sulfide-induced damage.

Other defects identified on the machined material surface include deep wear near the presence of very hard carbides, such as iron and chromium carbides. Carbides often do not fully remove during cutting and can cause local damage, irregularities, and wear on the machined surface as well as tool damage or cracking. Carbides may also be pulled out by the tool, leaving voids or depressions at their location (Figure 9). EDS analysis confirmed that the black area on the milled surface after 60 min of tool operation (Figure 9) is carbide precipitation (Figure 9a,c). Analysis of the voids near the carbide—region 3—revealed the presence of Al, Ti, and Si, elements that are part of the nanocomposite coating nACo3 (Figure 9a,d). This indicates that the tool was damaged due to contact with the hard sulfide. Sulfides were also observed on the machined steel surface (Figure 9e). Similar observations were made by the authors in the publications [42,43], who described the correlation between inclusions on the machined material surface and the occurrence of damage and defects in the machined surface.

**Figure 10:**
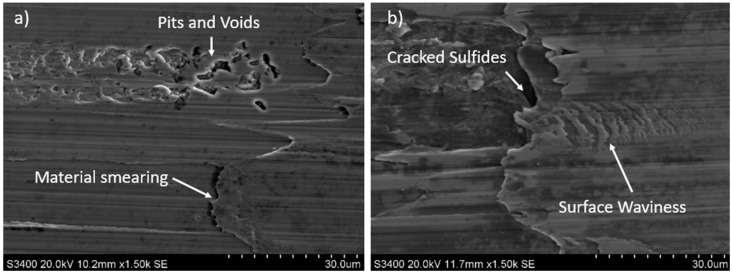
SEM images of 316L stainless steel surface showing (**a**) pitting and depressions after 15 min and (**b**) visible surface waviness after 20 min of tool operation.

The machined material surface also exhibited small, irregular indentations, often near inclusions or material imperfections (Figure 10a), as well as waviness on the machined material surface.

## 4. Comparison with Previous Studies

The results obtained in this study show a clear improvement in both tool life and surface quality when using the nanocomposite nACo3 (AlTiSiN) coating. These findings are consistent with previous research by He et al. [13], who reported enhanced performance of AlTiN-coated tools during dry machining of stainless steel. However, our results demonstrate an even more pronounced reduction in flank wear, likely due to the presence of silicon in the nACo3 structure, which contributes to grain refinement and improved oxidation resistance.

A comparison with the study by Maruda et al. [12] reveals similar trends in the relationship between feed rate and surface roughness. While their work focused on TiAlN coatings, they also observed increased roughness with higher feed rates. Our results confirm this trend but show slightly lower roughness values at comparable feed rates, which may be attributed to the superior tribological properties of the AlTiSiN coating used in our study.

Furthermore, the work of Carvalho et al. [34] emphasized the influence of coating composition on tool wear behavior during the machining of austenitic stainless steels. Their observations regarding oxidation and adhesion wear mechanisms are consistent with our SEM and EDS analyses, which confirm diffusion-related wear processes at elevated cutting speeds.

Compared to the multilayer coatings investigated by Gaurav [11], the single-layer nanocomposite structure evaluated in our research appears to offer more stable performance across a wider range of cutting parameters, particularly in terms of maintaining surface integrity and reducing built-up edge formation.

In summary, while our findings align with existing research in terms of general trends, they also provide novel insights by evaluating a less commonly studied coating type (AlTiSiN nanocomposite) and combining surface roughness and wear analysis in the context of machining 316L stainless steel under varied conditions.

## 5. Conclusions

The conducted studies showed that the application of PVD coatings, particularly the nanocomposite nACo3 coating (AlTiSiN), significantly improves the tool life and surface quality after milling of 316L stainless steel under air cooling conditions. The nACo3 coating effectively reduces the intensity of tool wear, extending tool life and improving cutting process stability.

The best results were achieved for cutting times up to 90 min, after which a sharp increase in tool wear and deterioration of surface quality occurred. After 90 min, clear signs of wear were observed, and after 120 min, the cutting process became inefficient due to increased cutting forces, intensive wear of the tool’s cutting edge, and significant degradation of the machined surface. Roughness measurements confirmed that the lowest values of Sa, Ra, and Rz parameters were achieved during the initial cutting phase; however, after exceeding the optimal time, the tool began to lose its cutting properties, negatively impacting surface quality.

Additional SEM and EDS analyses revealed that a critical issue in machining 316L stainless steel is the material adhering to the tool, which can lead to Build-Up Edge (BUE) and increased friction, causing higher temperatures and intensified wear. Moreover, the presence of sulfide inclusions in the steel’s microstructure contributed to local damage on both the machined surface and the tool, further shortening its lifespan. It was found that during the initial phase of machining, the surface quality improved due to tool break-in, but extended cutting time led to excessive heating and increased cutting forces, resulting in surface deformations and burns.

The findings underscore the importance of selecting appropriate cutting parameters, tool geometry, and cooling strategies for optimizing the machining process. Properly chosen PVD coatings, such as nACo3, can significantly enhance tool wear resistance, but controlling cutting conditions is still necessary to minimize negative effects such as excessive heating and friction.

Further research should focus on analyzing the impact of different cutting speeds, lubrication strategies, and new generations of PVD coatings on improving the efficiency of machining 316L stainless steel. It is also worthwhile to investigate alternative cooling methods, such as minimum quantity lubrication (MQL) or cryogenic cooling, which may further reduce tool wear and improve the quality of the machined surface.

316L stainless steel is austenitic and does not undergo martensitic transformation under normal conditions. The cryogenic process can be applied in some specialized applications, such as surgical tools, aerospace components, or precision mechanical parts, where minimizing deformation and improving dimensional stability are crucial.

In conclusion, the results clearly indicate that the application of PVD coatings, such as nACo3, is an effective method for improving the durability of cutting tools and the quality of the machined surface, but it requires proper optimization of process parameters to maximize both technological and economic benefits.

## Figures and Tables

**Table 1:** Comparison of selected PVD coatings applied to cutting tools for stainless steel machining: coating type, substrate, mechanical properties, and application performance.

Coating Type	Tool Material	Workpiece Material	Machining Method	Coating Parameters		
				Nano Hardness [GPa]	Dry Friction Coefficient µ	Maximum Working Temperature [°C]
AlCrN [11]	Carbide	316L	Milling	32–34	0.4	1100
	The AlCrN coating has excellent resistance to high temperatures and thermal shocks (up to 1100 °C). The presence of chromium increases the resistance to oxidation and also improves the mechanical strength of the coating. AlCrN is an ideal choice for machining with high mechanical and thermal loads, especially in impact cutting operations. In working conditions with or without cooling, it provides exceptional tool life.					
TiCN [13]	Carbide	304	Turning	28–30	0.3	500
	TiCN is a coating that is an extension of classical TiN. The addition of carbon (C) to the TiN structure leads to the formation of a hard, abrasion-resistant titanium carbonitride, which has higher hardness than TiN and better tribological properties. TiCN-coated cutting tools show less damage to the cutting edges of the tools compared to other tested PVD—AlTiN coatings. Therefore, it has been shown that this system provides better and more efficient tool operation in this type of application.					
TiAlN [12]	Carbide	316L	Turning	32–33	0.5	800
	TiAlN coating is a classic and versatile PVD coating, often used on general purpose tools. In this study, the effect of various PVD coatings, including TiAlN and TiN, on the wear of cutting tools during rotation of 316L austenitic stainless steel was evaluated, and it was found that the lowest wear was observed with an 8-layer TiAlN coating at a ratio of 33/67%–TiN/TiAlN. The highest VB values were observed for the uncoated and TiN-coated inserts.					
TiN [23,24]	Carbide	304 i 420	Milling	24–26	0.4	600
	TiN is one of the most commonly used tool coatings due to its favorable cost-to-performance ratio. This paper investigates surface roughness and chip shapes when milling stainless steel (304 and 420) using minimal quantity lubrication (MQL) with a vegetarian fluid on uncoated/coated/TiN-coated WC cutting tools and compares the results to dry machining. The TiN and MQL coatings made the chips more regular and acceptable for both materials being machined. Surface roughness was improved when using TiN-coated tools and using MQL with a vegan fluid.					
AlTiN [13]	Carbide	316L	Milling	34	0.4	900
	The AlTiN coating contains a larger amount of aluminum than TiAlN, which affects the formation of a protective layer of aluminum oxide (Al_2_O_3_) during operation. This layer acts as a thermal barrier, significantly increasing resistance to oxidation and enabling operation at very high temperatures (up to about 900 °C). AlTiN works great in dry machining, especially in operations with high thermal intensity (e.g., milling or drilling stainless steel).					
nACo [30]	Carbide	316L	Milling	39–40	0.4	1200
	nACo is a coating from the AlTiSiN group. Due to the silicon (Si) content, an amorphous Si_3_N_4_ layer is created in the structure, which significantly increases the thermal and mechanical resistance of the coating. Thanks to the use of a nanocomposite structure and the addition of silicon, it offers very long tool life when working in the most difficult cutting conditions under high thermal loads—especially where other coatings fail.					

**Table 2:** General mechanical properties of AISI 316L stainless steel used as the workpiece material.

Specification	Typical Value
Density (g/cm^3^)	7.9
Young’s Modulus (GPa)	200
Hardness, Rockwell B	95
Tensile Strength (MPa)	485
Poisson’s Ratio	0.3
Elongation (%)	40
Fatigue Strength (MPa)	146

**Table 3:** Chemical composition of AISI 316L stainless steel in weight percentage (%wt).

C	Mn	Si	P	S	Cr	Ni	N	Mo
0.03	2.00	0.75	0.05	0.03	18.00	14.00	0.10	3.00

**Table 4:** Geometric parameters of the carbide end mills used in the experimental milling tests.

Parameter	Value	Test Tool
Coating	nACo3	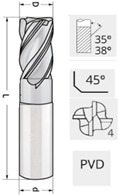
Shank diameter D [mm]	12	
Tool diameter d [mm]	12	
Tool length L [mm]	82	
Number of teeth z	4	
Helix angle β [°]	35–38	
Radial rake angle γ [°]	8	
Radial relief angle α [°]	6	
CER r_Ꜫ_ [μm]	8–10	
Corner	0.25 × 45°	

**Table 5:** Physical and mechanical properties of the WC–Co carbide substrate (MK12 grade) used in the tool manufacturing.

Grade	Grain Size (μm)	Cobalt Content (wt%)	Density (g/cm^3^)	Hardness HV30
MK12	0.18 (ultrafine)	12	14.2	1730

**Table 6:** Main properties and application range of the nACo3 (AlTiSiN) coating applied on the cutting tools.

Parameters	nACo3	Application
Nano-hardness [GPa]	39–41	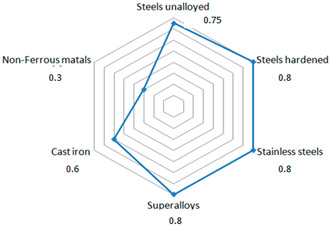
Coefficient of friction [μ]	0.4	
Coating thickness [μm]	3	
Max. Service temperature [°C]	1200	
Coating temperature [°C]	400–500	
Color	blue violet	

**Table 7:** The effect of cutting time on surface quality of 316L—SEM images at 100× (general surface topography) and 600× (local damage characters).

		Surface Photography After Machining at Magnification:	
		Mag. 100×	Mag. 600×
Tool life in the machining process, min	0	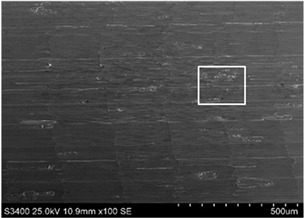	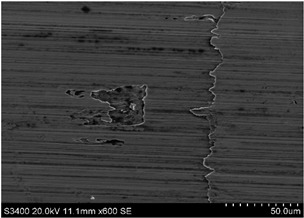
	30	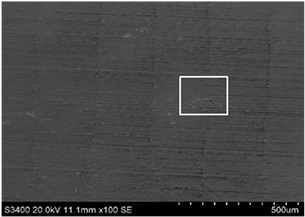	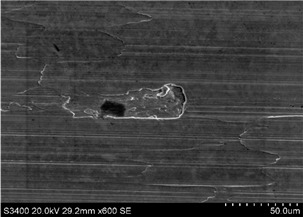
	60	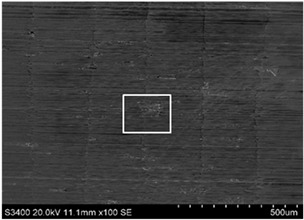	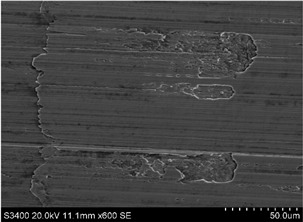
	90	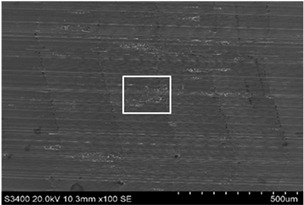	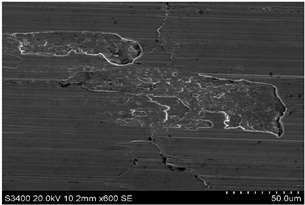
	120	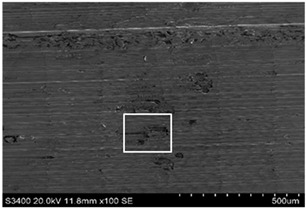	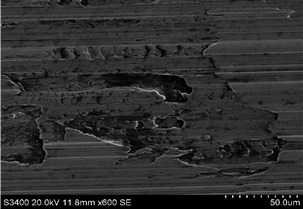

**Table 8:** Evolution of surface roughness parameters (Sa, Ra, Rz) of machined 316L stainless steel depending on tool usage time.

t [min]	Surface Map	Sa	Ra	Rz
		[µm]		
0	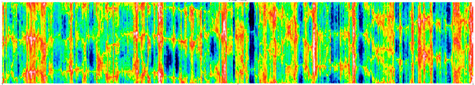	0.831	0.768	0.966
30	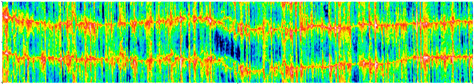	0.629	0.489	0.618
60	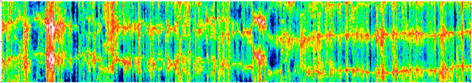	0.588	0.530	0.672
90	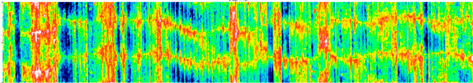	0.760	0.681	0.865
120	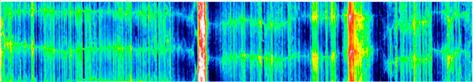	0.990	0.954	1.371

## Data Availability

The original contributions presented in this study are included in the article. Further inquiries can be directed to the corresponding author.

## References

[1] Pusavec F., Kramar D., Krajnik P., Kopac J. (2010). Transitioning to Sustainable Production—Part II: Evaluation of Sustainable Machining Technologies. J. Clean. Prod..

[2] Biermann D., Iovkov I. (2015). Modelling, Simulation and Compensation of Thermal Effects for Complex Machining Processes. Prod. Eng..

[3] Zębala W., Kowalczyk R. (2015). Estimating the Effect of Cutting Data on Surface Roughness and Cutting Force during WC-Co Turning with PCD Tool Using Taguchi Design and ANOVA Analysis. Int. J. Adv. Manuf. Technol..

[4] Liu J., Ma C., Tu G., Long Y. (2016). Cutting Performance and Wear Mechanism of Sialon Ceramic Cutting Inserts with TiCN Coating. Surf. Coat. Technol..

[5] Tu G., Wu S., Liu J., Long Y., Wang B. (2016). Cutting Performance and Wear Mechanisms of Sialon Ceramic Cutting Tools at High Speed Dry Turning of Gray Cast Iron. Int. J. Refract. Met. Hard Mater..

[6] Kupczyk M., Jozwiak K., Cieszkowski P., Libuda P. (2007). Influence of Laser Heating on Adhesion of CVD Coatings to Cutting Edges. Surf. Coat. Technol..

[7] Mikołajczyk T., Nowicki K., Kłodowski A., Pimenov D.Y. (2017). Neural Network Approach for Automatic Image Analysis of Cutting Edge Wear. Mech. Syst. Signal Process..

[8] Mikołajczyk T., Nowicki K., Bustillo A., Pimenov D.Y. (2018). Predicting Tool Life in Turning Operations Using Neural Networks and Image Processing. Mech. Syst. Signal Process..

[9] Jackson M.J., Morrell J.S. (2015). Machining with Nanomaterials.

[10] Grzesik W. (1998). Podstawy Skrawania Materiałów Metalowych.

[11] Gaurav S., Sargade V.G. (2011). Comparative Performance Evaluation of Uncoated and Coated Carbide Inserts in Dry End Milling of Stainless Steel (SS 316L). Int. Conf. Comput. Intell..

[12] Maruda R.W., Szczotkarz N. (2016). The Influence of the Type of Coating on the Cutting Tool Wear During Turning of 316L Austenitic Stainless Steel. Arch. Mech. Technol. Mater..

[13] He Q., Paiva J.M., Kohlscheen J., Beake B.D., Veldhuis S.C. (2019). An Integrative Approach to Coating/Carbide Substrate Design of CVD and PVD Coated Cutting Tools during the Machining of Austenitic Stainless Steel. Ceram. Int..

[14] Zlámal T., Mrkvica I., Szotkowski T., Malotova S. (2019). The Influence of Surface Treatment of PVD Coating on Its Quality and Wear Resistant. Coatings.

[15] Saini M., Sharma R., Abhinav, Singh G., Mangla P. (2016). Optimizations of Machining Parameter in Wire EDM for 316L Stainless Steel by Using Taguchi Method, ANOVA, and Grey Analysis. Int. J. Mech. Eng. Technol. (IJMET).

[16] Szczotkarz N., Mrugalski R., Maruda R., Królczyk G., Legutko S., Leksycki K., Dębowski D., Pruncu C. (2021). Cutting Tool Wear in Turning 316L Stainless Steel in the Conditions of Minimized Lubrication. Tribol. Int..

[17] Franczyk E., Małek M. (2023). Empirical Study on the Effect of Tungsten Carbide Grain Size on Wear Resistance, Cutting Temperature, Cutting Forces and Surface Finish in the Milling Process of 316L Stainless Steel. Adv. Sci. Technol. Res. J..

[18] Maranhão C., Davim J. (2010). Finite Element Modelling of Machining of AISI 316 Steel: Numerical Simulation and Experimental Validation. Simul. Model. Pract. Theory.

[19] Arrazola P.J., Arriola I., Davies M.A. (2009). Analysis of the Influence of Tool Type, Coatings, and Machinability on the Thermal Fields in Orthogonal Machining of AISI 4140 Steels. CIRP Ann. Manuf. Technol..

[20] Umbrello D., M’Saoubi R., Outeiro J. (2007). The Influence of Johnson–Cook Material on Finite Element Simulation of Machining of AISI 316L Steel. Int. J. Mach. Tools Manuf..

[21] Arrazola P.J., Ugarte D., Domínguez X. (2008). A New Approach for Friction Identification during Machining through the Use of Finite Element Modelling. Int. J. Mach. Tools Manuf..

[22] Uysal A., Demiren F., Altan E. (2016). Investigation of Surface Roughness and Chip Forms in Milling of Stainless Steel by MQL Method. Acta Phys. Pol. A.

[23] Khana M.M.A., Mithua M.A.H., Dharb N.R. (2009). Effects of Minimum Quantity Lubrication on Turning AISI 9310 Alloy Steel Using Vegetable Oil-Based Cutting Fluid. J. Mater. Process. Technol..

[24] Sharma J., Sidhu B.S. (2014). Investigation of Effects of Dry and Near Dry Machining on AISI D2 Steel Using Vegetable Oil. J. Clean. Prod..

[25] Lv D., Yu X., Wang Y. (2023). Evaluation of Cutting Edge K Form Factor in Milling of 316L Stainless Steel: A Study Based on FEM. Int. J. Adv. Manuf. Technol..

[26] Grabowski M., Małek M., Teimouri R., Skoczypiec S. (2024). Effect of Cutting-Edge Geometry on the Machinability of 316L Austenitic Steel. Adv. Sci. Technol. Res. J..

[27] Ibrahim R., Long C.Y., Ng Y., Rafai N.H., Mustapa M.S., Ismail A.E. (2021). Experimental Study on the Degree of Surface Generation by Edge Preparation Tools in Milling 316L. Int. J. Eng. Technol..

[28] Ozcelik B., Kuram E., Simsek B.T. (2011). Comparison of Dry and Wet End Milling of AISI 316 Stainless Steel. Mater. Manuf. Process..

[29] Ciftci I. (2006). Machining of Austenitic Stainless Steels Using CVD Multi-Layer Coated Cemented Carbide Tools. Tribol. Int..

[30] Polcar T., Cavaleiro A. (2011). High-Temperature Tribological Properties of CrAlN, CrAlSiN and AlCrSiN Coatings. Surf. Coat. Technol..

[31] Natesh C.P., Shashidhara Y.M., Amarendra H.J., Shetty R., Harisha S.R., Shenoy P.V., Nayak M., Hegde A., Shetty D., Umesh U. (2023). Tribological and Morphological Study of AISI 316L Stainless Steel during Turning under Different Lubrication Conditions. Lubricants.

[32] Haršáni M., Sahul M., Zacková P., Caplovič Ľ. (2017). Study of Cathode Current Effect on the Properties of CrAlSiN Hard Coatings Deposited by LARC. Vacuum.

[33] Ding X., Zeng X.T., Liu Y.C. (2011). Structure and Properties of CrAlSiN Nanocomposite Coatings Deposited by Lateral Rotating Cathode Arc. Thin Solid Films.

[34] Carvalho S., Ribeiro E., Rebouta L., Pacaud J., Goudeau P., Renault P.O., Rivière J.P., Tavares C.J. (2003). PVD Grown (Ti,Si,Al)N Nanocomposite Coatings and (Ti,Al)N/(Ti,Si)N Multilayers: Structural and Mechanical Properties. Surf. Coat. Technol..

[35] Cselle T., Coddet O., Galamand C., Holubar P., Jilek M., Jilek J., Luemkemann A., Morstein M. (2009). TripleCoatings3^®^–New Generation of PVD-Coatings for Cutting Tools. J. Mach. Manuf..

[36] Zhang Z., Liu Z., Ren X., Zhao J. (2023). Prediction of Tool Wear Rate and Tool Wear during Dry Orthogonal Cutting of Inconel 718. Metals.

[37] Zawada-Michalowska M., Pieśko P., Józwik J. (2020). Tribological Aspects of Cutting Tool Wear during the Turning of Stainless Steels. Materials.

[38] Caldeirani Filho J. (2002). Influence of Cutting Conditions on Tool Life, Tool Wear and Surface Finish in the Face Milling Process. J. Braz. Soc. Mech. Sci..

[39] Byrne G., Dornfeld D., Denkena B. (2003). Wear Mechanisms in Cutting Tools, Advancing Cutting Technology. CIRP Ann..

[40] Astakhov V. (2006). Surface Integrity in Machining Stainless Steel. Tribology of Metal Cutting.

[41] Liu G., Zou B., Huang C., Wang X., Wang J., Liu Z. (2016). Tool Damage and Its Effect on the Machined Surface Roughness in High-Speed Face Milling the 17-4PH Stainless Steel. Int. J. Adv. Manuf. Technol..

[42] Maciejewski J. (2015). The Effects of Sulfide Inclusions on Mechanical Properties and Failures of Steel Components. J. Fail. Anal. Preven..

[43] Rieders N., Nandasiri M., Mogk D., Avci R. (2021). New Insights into Sulfide Inclusions in 1018 Carbon Steels. Metals.

